# Book Reviews

**Published:** 1917-11

**Authors:** 


### BOOK REVIEWS

**Books mentioned may be inspected at and ordered through this office. So far as possible, books received in any month will be reviewed in the issue of the second month following. Pamphlets, quarterly and similar periodicals, reports, transactions, etc., will, as a rule, merely be mentioned.**

---

**The Prescription.** Otto A. Wall, Ph. G. M., M. D., St. Louis; C. V. Mosby Co., St. Louis. 4th revised edition, 274 pages, $2.50.

Under general considerations, the author discusses the Galenicals and various special points, as the distinction among officinal, official and pharmacopoeial. Weights and Measures include Oldberg's proposed method of making the drachm correspond to exactly 4 grams and the grain to 1/16 of a gram and also take up the matter of prescribing by percentages and proportions and of approximate measures. Under the head of Language, various full lists of Latin terms, abbreviations, etc., are given. The extemporaneous prescription is dealt with at length. The most interesting, if not the most practical part of the work is devoted to history and superstitions and shows a surprising amount of scholarly research.

---

**Diseases of the Skin.** Richard L. Sutton, M. D., Kansas City; C. V. Mosby Co., St. Louis. 2d revised edition, 1021 pages, 833 illustrations, 8 colored plates, $6.50.

The author adheres as rigidly as possible to the pathologic classification: hyperaemia, inflammation, haemorrhage, hypertrophy, atrophy, pigmentation anomalies, neuroses, new growths. Unlike some authors, and especially notable from his experience as a naval surgeon, he does not give an undue consideration to syphilis but discusses its manifestations under the various pathologic headings. Some of the most interesting details are included under the appendages: hair and follicles, sebaceous glands, coil glands and nails; and the mucous membranes adjoining the skin. The naval experience alluded to, undoubtedly allows the author to speak from direct familiarity with a number of race tropic affections.

---

**The Child in Health and Illness.** Dr. Carl G. Leo-Wolf, Buffalo, with an introduction on Prenatal Care by Dr. Peter W. Van Peyma, Buffalo. George H. Doran Co., N. Y. 297 pages, 68 illustrations, mainly from photographs by Dr. E. A. Sharp of Buffalo, charts of weight, etc., $2.

While this book is expressly designated as a text book for mothers and wives, it deserves the consideration of the medical profession, largely because its particular object includes the discussion of a number of details, such, for instance, as the baby buggy, which do not usually find themselves in technical treatises and because the more technical details are adapted to ultimate clinical use. The scheme for the appearance of the teeth (page 32) is ingenious and more easily memorized than any other that we have seen. Feeding is based on a discussion of general principles, shows a practical familiarity with the many difficulties encountered, and is elucidated by a special chapter devoted to recipes. The care of the infant, both as to feeding, clothing, education and other points, is carried well beyond the earliest stages. Defects of the Eye and Blindness are discussed in a special chapter by F. Parke Lewis, M. D., of Buffalo. We feel especially proud that so timely and practical a treatise should represent the professional scholarship of our own city.

